# Type of screen time moderates effects on outcomes in 4013 children: evidence from the Longitudinal Study of Australian Children

**DOI:** 10.1186/s12966-019-0881-7

**Published:** 2019-11-29

**Authors:** Taren Sanders, Philip D. Parker, Borja del Pozo-Cruz, Michael Noetel, Chris Lonsdale

**Affiliations:** 1 Institute for Positive Psychology and Education, Australian Catholic University, North Sydney, NSW Australia; 2 School of Behavioural and Health Sciences, Australian Catholic University, Brisbane, QLD Australia

**Keywords:** Screen time, Children, Health, Education

## Abstract

**Background:**

Excessive engagement with digital screens is harmful to children’s health. However, new evidence suggests that exposure at moderate levels may not be harmful and may even provide benefit. Therefore, our objective was to determine if there are curvilinear relationships between different types of screen time and a diverse set of outcomes, including health and education.

**Methods:**

We address our objective using a repeated measures design. Children (*N* = 4013), initially aged 10–11 were assessed every 2 years between 2010 and 2014. Children’s screen time behavior was measured using time-use diaries, and categorized into five types: social, passive, interactive, educational, or other. We used measures of children’s physical health, health-related quality of life, socio-emotional outcomes, and school achievement. The analysis plan was pre-registered. Models were adjusted for gender, socio-economic status, ethnicity, number of siblings, and housing factors.

**Results:**

There were linear associations between total screen time and all outcomes, such that more screen time was associated with worse outcomes. However, there was variability when examined by screen time type. Passive screen time (e.g., TV) was associated with worse outcomes, educational screen time (e.g., computer for homework) was associated with positive educational outcomes and had no negative relations with other outcomes. Interactive screen time (e.g., video games) had positive associations with educational outcomes but negative associations with other outcomes. In all instances, these significant associations were small or very small, with standardised effects < 0.07. We found little evidence of curvilinear relationships.

**Conclusions:**

The small effects of screen time on children’s outcomes appear to be moderated by the type of screen time. Policy makers, educators, and parents should consider the type of screen time when considering the benefits and harms of use.

## Background

High levels of engagement with digital screens (i.e., ‘screen time’) are harmful to children’s physical health [1]. A body of evidence underpins guidelines that recommend limiting children’s screen time exposure [2, 3]. For example, a recent review found that screen time is deleteriously associated with adiposity and cardiorespiratory fitness [1]. There is also evidence that screen time is associated with negative psychological and educational outcomes, such as greater depression [4] and lower academic achievement [5], respectively. As a result, guidelines [3, 6] advise that lower levels of screen time are associated with benefits for children. In our study, we refer to this as the *less-is-better hypothesis*.

Evidence that moderate levels of screen time may have benefits over abstinence or high use contradicts current guidelines. For example, a review of the literacy development literature revealed studies in which moderate amounts of television was associated with better reading than low or high amounts of viewing [7]. Curvilinear relations have also been found with psychosocial outcomes. For example, in an investigation of more than 120,000 adolescents, Przybylski and Weinstein found moderate amounts of electronic screen time were associated with higher mental well-being compared to low or high levels [8]. Similar curvilinear relationships for screen time have also emerged in other studies related to children’s health and well-being [9–12]. Some researchers have labeled this the *Goldilocks hypothesis* [8].

When examining the Goldilocks screen hypothesis, previous studies have tended to focus on a single outcome, or a narrow range of variables. For example, Przybylski and Weinstein (2017) centred their investigation on screen time’s association with adolescents’ well-being, and did not investigate other important outcomes, such as physical health or educational achievement. It is possible that the less-is-better hypothesis and the Goldilocks hypothesis apply differentially to outcomes. For example, engaging with moderate amounts of social media may benefit social functioning, while high levels might displace face-to-face contact, leading to poorer mental health (i.e., supporting the Goldilocks hypothesis) [13]. In contrast, passive screen time (e.g., television) would be unlikely to convey any form of physical health benefit, and thus lower levels would be expected to provide health benefits (i.e., supporting the less-is-better hypothesis). Studies that examine a limited range of outcome variables [8–12] cannot examine this possibility.

In the current study, we aimed to investigate these two competing hypotheses across different types of screen time and different outcomes, including physical health, psychological outcomes, and educational outcomes. We further extended the Przybylski and Weinstein (2017) study of adolescents by examining these hypotheses in a large sample of children, and by examining if these relationships are stable as children age. As this is a conceptual replication of Przybylski and Weinstein’s work, we also examine differences by weekday and weekend.

### Research Questions

1. Are there linear or curvilinear relations between screen time and children’s physical health, psychological outcomes, and educational outcomes? And, if curvilinear relations exist, at what duration of screen exposure do they become negative?
2. Are these relationships modified by age, screen time type (e.g., device or content), and weekday vs. weekend usage? If so, do these factors shift the turning point?

## Method

### Study design and sample

Data were drawn from Growing Up in Australia: The Longitudinal Study of Australian Children (LSAC), a population-based study which tracks two cohorts of children aged 0/1 years (B-cohort) and 4/5 years (K-cohort) every 2 years beginning in 2004. We used data from Waves 4–6 of the K-cohort (2010–2014; ages 10–15). The overall response rate was 62% in the K-cohort (*N* = 4013) at baseline, with Wave 6 retention rates of 82%. Other waves of the K-cohort could not be included because of significant changes in the design of the time-use diary instrument used as our exposure measure [14]. We excluded the B-cohort because of the limited availability of time-use data during the ages of interest. Further details on the LSAC methodology, including sampling procedures, are available elsewhere [15].

### Exposure variables

#### Screen time

Time spent engaging with screens was measured using time-use diaries administered to the child. Children recorded the activities they participated in during one randomly allocated day on a paper diary. During a face-to-face interview on the day following the diary, an interviewer added additional contextual information (e.g., where they were and who they were with). Participants nominated the primary activity they engaged in, and the time of the activity (the activity ‘window’). The child could also nominate additional secondary behaviors that occurred in parallel during the activity window. The interviewer applied a coding framework to the children’s activities to make the diaries comparable across children [14]. We divided diary activities which involved screens into five categories: social screen time (e.g., social media), passive screen time (e.g., television), interactive screen time (e.g., video games), educational screen time (e.g., computer use for homework), and other screen time where the activity did not fit into any of the categories.

To process the time-use diaries, we calculated the total length of activities which represented screen time regardless of whether they were primary or secondary activities. To calculate total screen time, we added all activities windows where any of the activities included screen time. For example, if a child spent 15 min texting (primary) while also watching TV (secondary), then we calculated 15 min of both ‘social screen time’ and ‘passive screen time’, but only 15 min of ‘total screen time’ to avoid double-counting. Thus, it should be noted that the individual categories of screen time variables will not sum to total screen time. A list of items coded as screen time is provided in Additional file 1: Table S1. Time-use diaries have been successfully used in previous studies investigating health behaviours in children [16–20].

### Physical health

#### Physical outcomes

Weight was measured to the nearest 50 g using glass bathroom scales (Salter Australia, Springvale, VIC, Australia; Code 79985) while children were in light clothing. Height was measured twice, without shoes, to the nearest 0.1 cm using a stadiometer (Invicta, Leicester, UK; Code IPO955). Waist circumference was also assessed twice to the nearest 0.1 cm. Body mass index (BMI) was then calculated as kg/m^2^. The child’s BMI z-score for age was calculated based on Centre for Disease Control growth charts [21, 22]. All anthropometric measures were taken by the trained interviewer.

#### Global health

Parents were asked to report on their perception of their child’s overall health in a scale ranging from “poor” to “excellent” [23]. This scale has been previously validated for Australian children [24]. Because there were fewer than 20 children with “poor” or “fair” health, global health was dichotomized to “excellent” and “less than excellent”.

### Psychological outcomes

#### Social and emotional functioning

Children’s socio-emotional outcomes were assessed using the Strengths and Difficulties Questionnaire (SDQ), a validated, 25-item, parent-reported questionnaire [25]. We used all five subscales (conduct problems, emotional problems, hyperactivity, peer problems, and prosocial behavior; range: 0–10).

Children’s quality of life was assessed via the Paediatric Quality of Life Inventory (PedsQL), a validated 23-item parent-reported instrument [26]. We computed two subscale scores (social and emotional functioning), which ranged from 0 to 100. We chose not to include the physical functioning subscale as the items were unlikely to be related to screen time. A higher PedsQL score represents better quality of life. Parents were the respondents for both the SDQ and PedsQL.

#### Temperament profile

Children’s temperament was assessed with the School-Age Temperament Inventory, a 38-item parent-reported questionnaire with four dimensions: negative reactivity (intensity and frequency of negative affect), task persistence (the self-direction that a child exhibits in fulfilling tasks), approach/withdrawal (response to new people and situations), and activity (moves quickly to get where he/she wants to go) [27]. In the context of this study, only negative reactivity and task persistence were included because of their plausibility as outcomes of screen time. Higher scores indicate that the child is higher in negative reactivity and task persistence.

### Educational outcomes

#### School achievement

Estimates of both numeracy and literacy ability were taken from government administration records of the National Assessment Program - Literacy and Numeracy (NAPLAN, https://www.nap.edu.au/naplan). The NAPLAN data are linked to child data by the LSAC organisers via a unique identifier. The NAPLAN tests are given to all eligible children in Australia in Grades 3 (age 8), 5 (age 10), 7 (age 12), and 9 (age 14). We used scores from Grades 5–9. The tests are scaled so they are comparable across age cohort and across grade. Scores have an overall mean of 500 and a standard deviation of 100. Numeracy was measured using a single test and literacy was measured using four tests covering reading, writing, spelling, and grammar. We conducted principal component analysis on the the four literacy scores and formed a single factor score to represent literacy.

### Adjustment variables

To provide an all-else-being-equal estimate of the effect of screen time, we adjusted results for: child gender, Indigenous status, language-other-than-English status, child’s country of birth (Australia vs. elsewhere), and a composite measure of family socioeconomic status provided by the LSAC organizers [28], which is calculated using parent’s occupational prestige, income and education. We also used a measure of the average socioeconomic status of the child’s postcode [29]. To adjust for opportunity to engage in activities other than screen time, we further adjusted for home type (detached house vs other), number of siblings of the study child, and a parent-reported index of neighborhood livability (including parks and safety), as neighborhood factors have previously been linked to screen time [17].

### Analysis

To minimise potential bias we pre-registered our analysis plan prior to commencing the study, including specifying what analyses would be included and our criteria for including variables in the analysis [30]. Any deviations from the pre-registered plan are noted below. Analysis was based on Przybylski and Weinstein’s [8] study of screen time and well-being that provided support for the Goldilocks hypothesis. We fitted screen time as both linear and quadratic effects. If the quadratic effect was significant, we calculated the turning point (i.e., the point at which more screen time moved from having a beneficial to negative influence) using the equation: ${\hat{x}}_{max}=\frac{-{\beta}_{screen. time}}{2\times {\beta}_{screen. time. quadratic}}$. We also calculated the point at which increases in screen time led to poorer outcomes than no screen time calculated as twice the turning point, which we refer to as the ‘zero point’.

The LSAC data comes from a complex sampling design with postcode as the primary sampling unit. In addition, we combined data from different waves, meaning that each participant had multiple waves of data. To account for these factors, we used multilevel models with observations nested within individuals and individuals nested within postcodes. Our repeated measures design takes advantage of the multiple waves of data, but we do not test for longitudinal associations. We accounted for attrition by using all available information for each participant and using sample attrition weights provided by the survey organizers to ensure that the data remained representative of the population at each wave. We handled unit non-response missing data using multiple imputations, combining effects across 10 imputations [31]. We reverse coded variables such that increases could be consistently interpreted as improvements in these outcomes.

We tested unadjusted models and adjusted models. As we were interested in whether the effects differed by age or weekday versus weekend, all models included terms for age and weekday/weekend. Note that our pre-registered analysis plan [30] mistakenly included gender as both an interaction term and a control variable, and we chose to only include it as a control variable.

## Results

### Participants

Our analysis included 4013 children in the LSAC study. Of those analyzed, 51.2% were male, 96.1% were non-indigenous, 85.7% spoke English as their primary language, and 95.9% were born in Australia. Most children lived in a detached house (88.2%), and the study children had a mean of 1.7 siblings (SD = 1.2). At age 10 there were 4013 participants. This declined to 3682 by age 12 and 3276 by age 14. There was a notable increase in children’s educational and social screen time between the ages of 12 and 14. This increase may be due to the participants transitioning from primary to secondary schooling. Australian children typically begin high school at age 13, and this transition may increase their autonomy or change the amount of technology they use at school. Further description of the sample is found in Table 1. For unit non-response, the most missing data was for the time-use diaries (21%) and NAPLAN scores (16%). All other variables had less than 5% missing data (see Additional file 4: Figure S1).

**Table 1:** Sample descriptive statistics

	Wave 4 (Age 10–11)	Wave 5 (Age 12–13)	Wave 6 (Age 14–15)
Total Screen Time (min/day)	76.6 (35.88)	76.77 (35.85)	97.44 (36.19)
Social Screen Time (min/day)	0.59 (4.03)	0.56 (3.97)	22.3 (34.23)
Educational Screen Time (min/day)	2.56 (10.3)	2.61 (10.36)	11.18 (23.79)
Passive Screen Time (min/day)	61.37 (35.36)	61.35 (35.3)	65.27 (40.76)
Interactive Screen Time (min/day)	26.43 (32.66)	26.8 (32.84)	28.7 (40.19)
Other Screen Time (min/day)	1.24 (6.39)	1.25 (6.41)	12.11 (25.74)
Emotional Functioning (PedsQL)	74.01 (16.49)	75.64 (17.1)	74.97 (18.23)
Social Functioning (PedsQL)	80.24 (18.58)	82.61 (17.86)	80.54 (18.32)
Prosocial Behaviour (SDQ)	8.5 (1.66)	8.3 (1.74)	8.06 (1.86)
Peer Relationship Problems (SDQ)	1.5 (1.71)	1.43 (1.65)	1.54 (1.67)
Emotional Symptoms (SDQ)	1.9 (1.95)	1.94 (1.95)	1.89 (1.98)
Hyperactivity (SDQ)	3.16 (2.36)	2.93 (2.32)	2.66 (2.23)
Conduct Problems (SDQ)	1.33 (1.48)	1.06 (1.42)	0.97 (1.41)
Reactivity (SATI)	2.3 (0.81)	2.38 (0.78)	2.32 (0.81)
Task Persistence	3.5 (0.89)	3.57 (0.85)	3.65 (0.86)
Body Mass Index z-score	0.37 (1.03)	0.35 (1.03)	0.37 (1.13)
Waist Circumference (cm)	66.76 (9.4)	71.89 (10.26)	75.14 (10.19)
Global Health	0.44 (0.5)	0.46 (0.5)	0.48 (0.5)
Literacy	0.02 (1)	0.05 (0.99)	0.05 (0.97)
Numeracy	0.01 (1)	0.04 (1)	0.05 (0.99)

All data presented as means (standard deviations). *PedsQL* Pediatric Quality of Life Inventory, *SDQ* Strengths and Difficulties Questionnaire, *SATI* School-Age Temperament Inventory

### Preliminary analysis

**Fig. 1:**
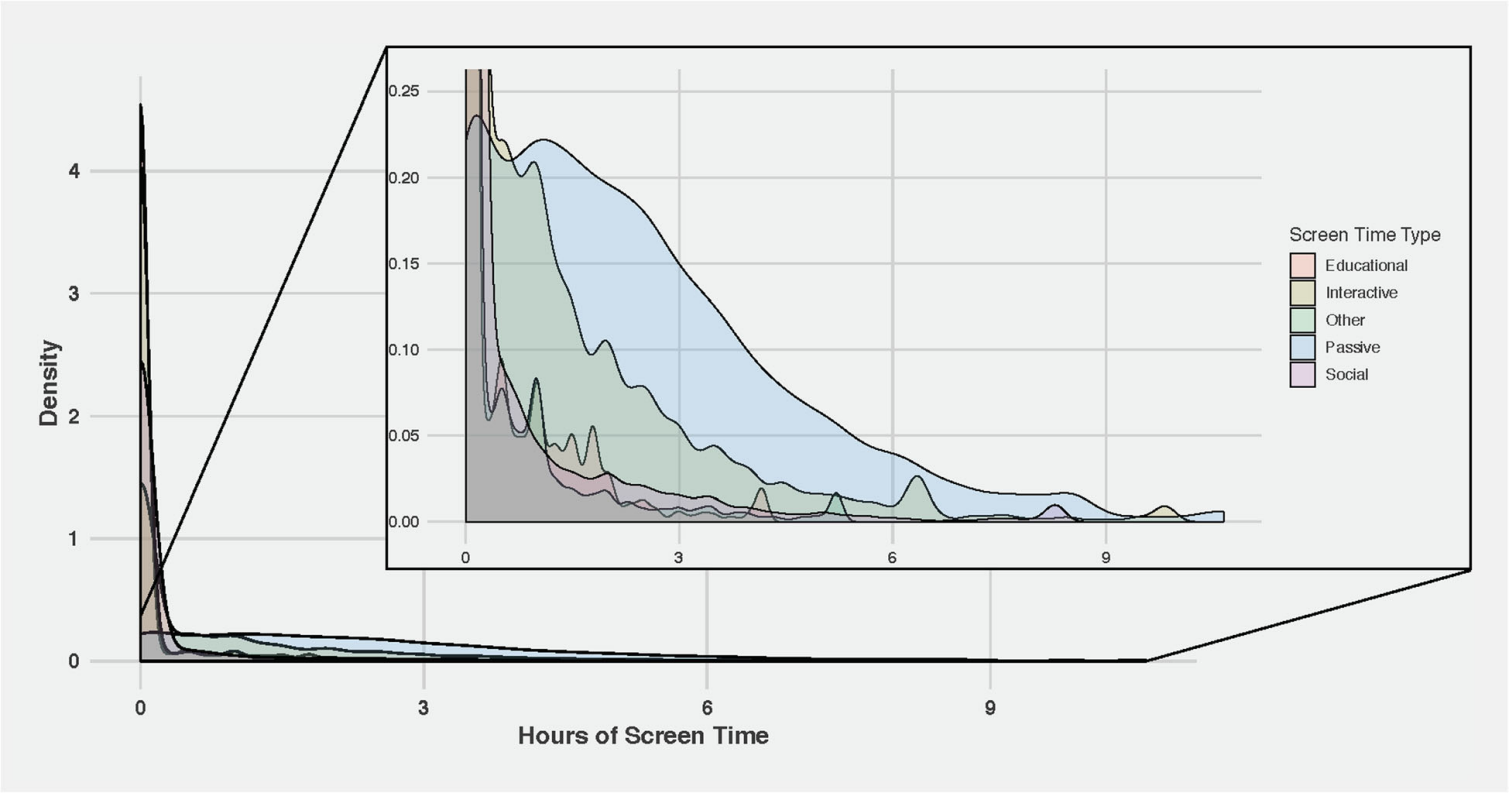
Density Plots for Components of Total Screen Time

Initial analyses showed that the screen time variables were positively skewed, especially for the less popular screen time types (e.g., social screen time) where there were high numbers of participants with zero screen time (Fig. 1). Therefore, we log transformed the screen time variables for imputation and translated back to the original scale for analysis. Despite evidence of skew in both exposure and some outcome variables, assumption checking revealed few problems in the models.

As per our pre-registered protocol [30], we checked that outcomes were independent using zero-order correlations and planned to remove variables if any were correlated above *r* = 0.70. The literacy and numeracy outcomes were correlated at *r* = 0.71 and we therefore created a composite score that was the unweighted mean of the first principal component of each of the two scores. We refer to this composite score as ‘school achievement’. We noted that PedsQL emotional subscale and SDQ emotional subscale were correlated at *r* = − 0.67 and BMI and waist circumference at *r* = 0.69 (see Additional file 2: Table S2 for other correlations). While these were below our *a priori* cutpoint, to minimize spurious associations we chose to keep only the variables with the least missing data (SDQ emotional subscale and waist circumference). We also checked for missing data prior to imputation, and planned to remove variables where missing data was > 60% [30]. No variables were removed on this basis.

### Linear effects

**Fig. 2:**
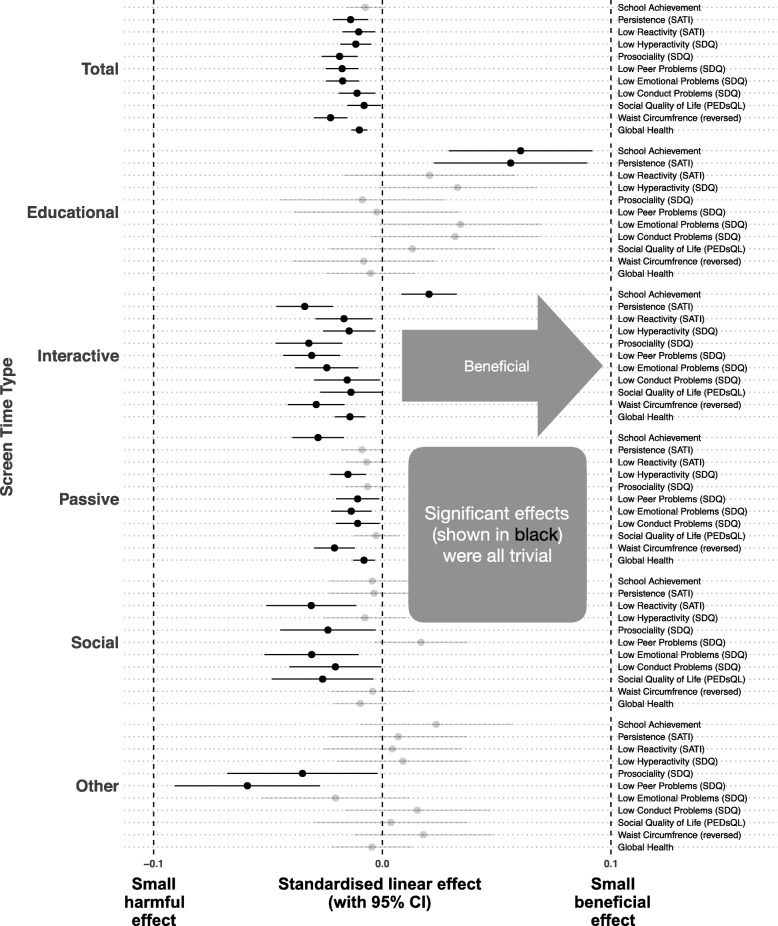
Adjusted Standardised Linear Effects for Each Combination of Screen Time Exposure and Outcome

To examine the less-is-better hypothesis, we first examined linear models without quadratic terms. These results are presented in Fig. 2 as adjusted linear effects, standardised for each outcome (β). These effects sizes are typically interpreted as small effect: β = 0.1; medium effect: β = 0.3; large effect: β = 0.5. All linear results were β < 0.07; that is, very small in size.

Total screen time was associated with worse educational outcomes, but this result was fully attenuated in adjusted models (see Additional file 3: Table S3). In both unadjusted and adjusted models, total screen time was linearly associated with unfavorable temperament outcomes, worse socio-emotional outcomes, lower health-related quality of life, and poorer health outcomes. However, there was substantial variability when results were examined by screen time type.

Educational screen time (e.g., homework on electronic devices) showed the most benefits in unadjusted and adjusted models, with positive effects on children’s persistence and educational outcomes, and no significant effects on psychological or health outcomes. Interactive screen time (e.g., video games) showed similar trends as total screen time. However, unlike total screen time, interactive screen time was associated with positive educational outcomes. Passive screen time (e.g., TV) was associated with worse psychological outcomes, poorer health outcomes, and lower educational outcomes in unadjusted and adjusted models. Poorer prosocial behavior and lower persistence were also associated with higher passive screen time, but only in unadjusted models. Social screen time was linearly associated with poorer health-related quality of life, higher reactivity, and worse socio-emotional outcomes for the prosocial, emotional, and conduct subscales of the SDQ, with no influence on the peer or hyperactivity subscales of the SDQ, nor children’s persistence, health, or educational outcomes. Finally, other screen time showed minimal associations with outcomes, with negative effects on only the prosocial and peer SDQ subscales.

### Quadratic effects

We next examined if any relationships were better represented by a quadratic function. In unadjusted models, there were non-linear relationships between total screen time and the hyperactivity SDQ subscale, social screen time and the peer SDQ subscale, interactive screen time and the hyperactivity and prosocial SDQ subscales and persistence, educational screen time and persistence, and other screen time and persistence. After adjustment for covariates, only the total screen time and hyperactivity SDQ subscale (β_Linear_ = 0.028 [0.013–0.043]; β_Quadratic_ = − 0.001 [0.002 – − 0.000]; turning point: 12.29 [6.44–18.14] hours; zero point: 24.59 [12.90–36.28] hours), and the social screen time and peer SDQ subscale (β_Linear_ = − 0.096 [− 0.159–0.034]; β_Quadratic_ = 0.011 [0.003–0.019]; turning point: 4.48 [3.42–5.53] hours, zero point: 8.96 [6.85–11.06] hours) quadratic associations remained significant (Additional file 7 Table S3). We note that, owing to the very small quadratic effect, the zero point for the total screen time and hyperactivity SDQ association is outside the range of plausible values. Scatterplots of all associations and the quadratic results are available in Additional file 5: Figure S2.

### Interactions with age and weekday

To determine the extent to which these relationships changed as the children aged, we tested an interaction between screen time and sample wave (as an indicator for age). There were very few significant interactions (6 of 132 for the linear effects and 3 of 132 for the quadratic effects in the adjusted models with *p* < .05), suggesting that these associations are stable between the ages of 10 and 15. All interaction results are available in Additional file 7: Table S3.

For the linear models, all six interactions related to age. Three interactions were present for waist circumference, and one each for prosociality, social PedsQL, and reactivity. All indicated that increased screen time had a more detrimental association with these outcomes at ages 10 and 12 than at age 14. No significant linear interactions were found for weekday vs weekend.

All three of the significant interactions for the adjusted quadratic relationships related to weekend vs weekday. The interactions were present for a) conduct problems, b) emotional problems, and c) reactivity as predicted by interactive screen time. All significant quadratic interactions indicated a Goldilocks effect for weekends, with turning points at approximately two to 3 h (see Additional file 6: Figures S3 and Additional file 7: Figure S4), and no quadratic effects on weekdays. No significant quadratic interactions were found for age.

## Discussion

In this study, we compared competing hypotheses for screen time effects on children’s physical health, psychological outcomes, and educational outcomes. We found evidence that screen time was associated with children’s physical health, health-related quality of life, socio-emotional outcomes, and school achievement, with substantial variation based on the type of screen time. In moderation analyses, these results appeared to remain stable for screen time on weekdays versus weekends. While there were some significant interactions, none were meaningful in terms of practical significance. There was little evidence to support the Goldilocks hypothesis in our data. Instead, our findings lend qualified support to the less-is-better hypothesis–qualified because educational screen time was associated with positive educational outcomes and higher persistence, with no negative consequences for other outcomes. Educational screen time, therefore, appears beneficial and would not fit the less-is-better or Goldilocks hypotheses. However, the magnitude of the effects observed in our study were consistently very small, with almost all less than 0.05 of a standard deviation per hour of additional screen time. This finding is consistent with meta-analytic results, where effect sizes for physical health [32] and socio-emotional and behavioral outcomes [33] have been small [34]. Yet, screen time has become a major concern that parents have about their children’s health [35]. Our results suggest that detrimental effects may be domain-specific and, as such, some of the concern around screen time may be unjustified.

Our results also demonstrate a need for future guidelines to embrace the complexity of screen time. We found that interactive screen time can be simultaneously harmful and beneficial, in that it negatively impacts most outcomes but is positively associated with educational outcomes. Most current guidelines [2, 3] focus on reducing harm and largely ignore the potential benefits some types of screen time can provide. Future evidence-based guidelines should focus on providing parents, and professionals who advise parents and children (e.g., doctors, teachers), with information that allows them to balance the risks and benefits of screen time. It is likely useful for parents to know that duration is not the only screen exposure variable to consider – content also matters. For example, our analyses show there are unlikely to be negative educational consequences, and there may even be some small benefits, when children engage in educational types of screen time such as using a computer for homework.

Our findings are in contrast to previous research that found non-linear relationships between screen time and mental well-being [8], socio-emotional outcomes [10], sleep [11], and other health outcomes [12]. One explanation is differences in sample sizes. For instance, Przybylski and Weinstein [8] investigated associations between different types of screen time and mental well-being in 120,000 adolescents. They found significant results, with standardized effect sizes for the quadratic terms between 0.03 and 0.13. It’s possible that even though our data included more than 10,000 data points, it was insufficient to detect these weak effects. If this is the case, we would question the clinical significance of such small effects.

### Strengths and limitations

We used a nationally-representative, longitudinal dataset, which provided time-use diary estimates of behavior, as opposed to simple recall questions. We preregistered our analysis plan prior to analyzing the data, and used methods to address the complex survey method and missing data. Finally, we examined a broad range of screen time exposures, including educational, interactive and passive forms of screen time. We also examined diverse outcomes, including physical health, psychological, and educational variables. In addition, we conditioned on a much broader range of potential covariates than previous research.

Despite these strengths, our study has several important limitations. As with the vast majority of screen time research [36], our study relied on subjectively-reported screen time. Currently, there are limited options for objectively measuring screen exposure. More precise measurement devices (e.g., wearable cameras) may yield more accurate determinations not only of screen exposure duration, but also the specific content being viewed. These measurement improvements may have less noise, and provide a clearer indication of the effects [37]. Despite using longitudinal data we would be reluctant to draw causal conclusions. The data used covers the period 2010–2014 and it is plausible that screen time behaviour has changed since these data were collected. As such, it is possible that the results presented here are not generalizable to contemporary children of the target ages. While we adjusted for important confounders there is still a risk of unmeasured variable bias influencing the findings (e.g., parenting style or companion) and we cannot rule out the possibility of reverse causation.

## Conclusion

Previous studies suggested that, compared with very low or very high amounts of screen time, moderate amounts of screen media use may benefit children’s mental well-being. Our findings contradict that research, with little support for the Goldilocks hypothesis across a wide range of physical health, psychological and educational outcomes. Indeed, we observed only very small effect sizes on the outcomes we measured and across the different types of screen time. We observed that what small effects do exist seem to be moderated by the type of screen time, with passive screen time (e.g., TV) having mostly detrimental effects, while educational screen time could confer slight benefits in school achievement and persistence. These results suggest that policymakers, professionals, and parents should consider the type of children’s screen time rather than just duration. However, our overall findings indicate that the high levels of concern about their children’s screen time exhibited by parents may be unwarranted.

## Supplementary information

**Additional file 1: Table S1.** Time Use Diary Categories.

**Additional file 2: Table S2.** Correlations Matrix.

**Additional file 3: Table S3.** Full Results.

**Additional file 4: Figure S1.** Missing Data.

**Additional file 5: Figure S2.** Scatterplots.

**Additional file 6: Figure S3.** Interactions and Quadratics.

**Additional file 7: Figure S4.** Example plot of unadjusted linear and quadratic effects: social quality of life predicted by social screen time with original scale (left) and modified scale (right).

## Data Availability

The LSAC dataset is available from the National Centre for Longitudinal Data (see https://growingupinaustralia.gov.au). The authors do not have permission to share this data without endorsement from the Australian Institute of Family Studies. Materials for this study, including analysis files and preregistered analysis plans, are available through the Open Science Framework (https://osf.io/bhzk8/).

## Abbreviations

BMI: Body Mass Index
LSAC: Longitudinal Study of Australian Children
NAPLAN: National Assessment Program - Literacy and Numeracy
PedsQL: Paediatric Quality of Life Inventory
SATI: School-Age Temperament Inventory
SDQ: Strengths and Difficulties Questionnaire
TV: Television
