# Supplementary material for: Type of screen time moderates effects on outcomes in 4013 children: evidence from the Longitudinal Study of Australian Children
Source: Int J Behav Nutr Phys Act. 2019 Nov 29;16:117. doi: 10.1186/s12966-019-0881-7 (PMC6884886; doi:10.1186/s12966-019-0881-7)
Supplement: Supplementary file 4 — Additional file 4: Figure S1. Missing Data. [file 12966_2019_881_MOESM4_ESM.pdf]

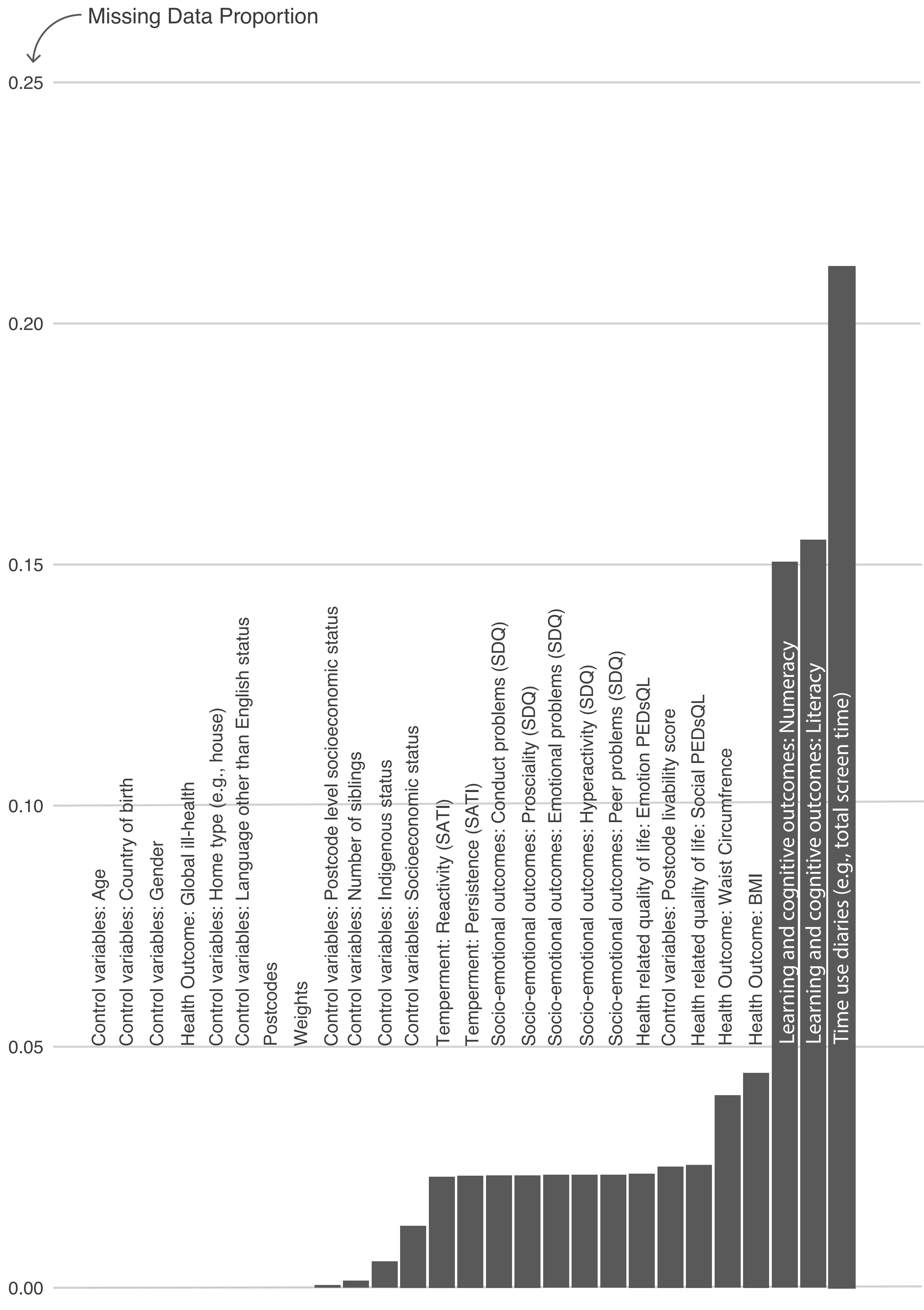
Missing Data Proportion
0.25
0.20
0.15
0.10
0.05
0.00
Control variables: Age
Control variables: Country of birth
Control variables: Gender
Health Outcome: Global ill-health
Control variables: Home type (e.g., house)
Control variables: Language other than English status
Postcodes
Weights
Control variables: Postcode level socioeconomic status
Control variables: Number of siblings
Control variables: Indigenous status
Control variables: Socioeconomic status
Temperment: Reactivity (SATI)
Temperment: Persistence (SATI)
Socio-emotional outcomes: Conduct problems (SDQ)
Socio-emotional outcomes: Prosciality (SDQ)
Socio-emotional outcomes: Emotional problems (SDQ)
Socio-emotional outcomes: Hyperactivity (SDQ)
Socio-emotional outcomes: Peer problems (SDQ)
Health related quality of life: Emotion PEDsQL
Control variables: Postcode livability score
Health related quality of life: Social PEDsQL
Health Outcome: Waist Circumfrence
Health Outcome: BMI
Learning and cognitive outcomes: Numeracy
Learning and cognitive outcomes: Literacy
Time use diaries (e.g., total screen time)
