# Supplementary material for: Type of screen time moderates effects on outcomes in 4013 children: evidence from the Longitudinal Study of Australian Children
Source: Int J Behav Nutr Phys Act. 2019 Nov 29;16:117. doi: 10.1186/s12966-019-0881-7 (PMC6884886; doi:10.1186/s12966-019-0881-7)

Supplementary Figure 2: Scatterplots with linear (coloured) and quadratic (black) effects for each exposure and outcome

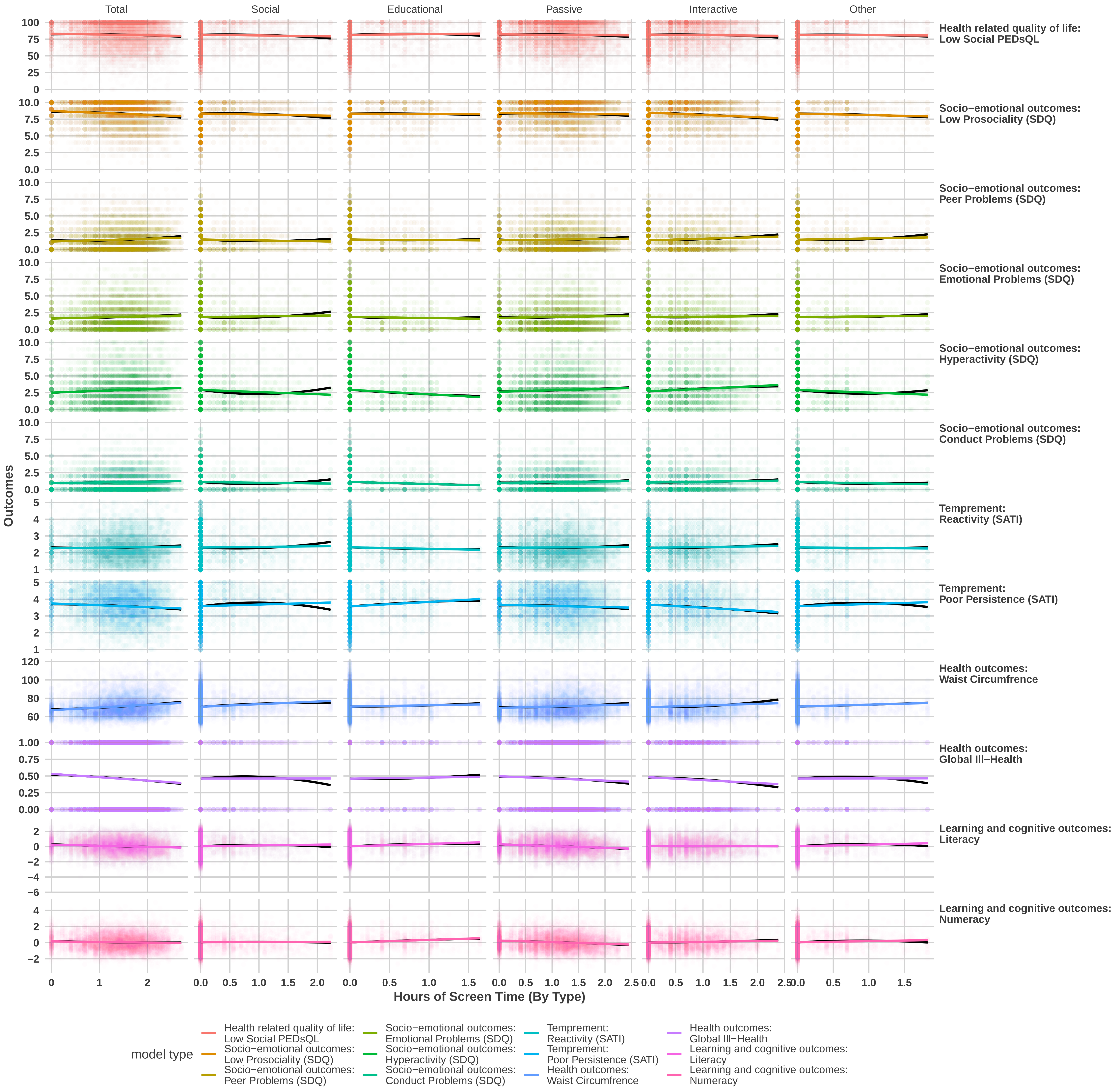

Supplement: Supplementary file 5 — Additional file 5: Figure S2. Scatterplots. [file 12966_2019_881_MOESM5_ESM.pdf]
