# Supplementary material for: Type of screen time moderates effects on outcomes in 4013 children: evidence from the Longitudinal Study of Australian Children
Source: Int J Behav Nutr Phys Act. 2019 Nov 29;16:117. doi: 10.1186/s12966-019-0881-7 (PMC6884886; doi:10.1186/s12966-019-0881-7)
Supplement: Supplementary file 6 — Additional file 6: Figure S3. Interactions and Quadratics. [file 12966_2019_881_MOESM6_ESM.pdf]

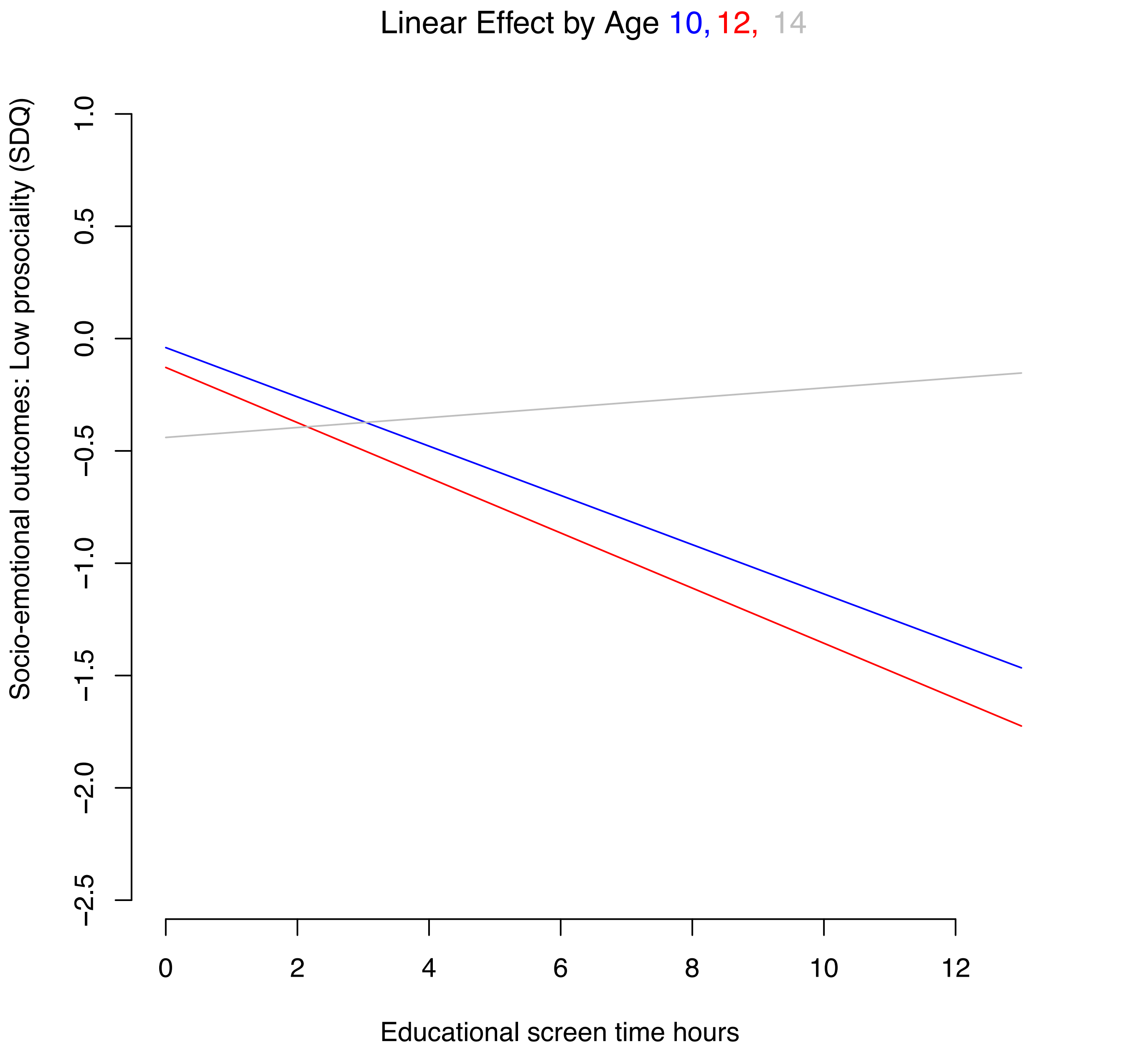
Linear Effect by Age 10, 12, 14
Socio-emotional outcomes: Low prosociality (SDQ)
1.0
0.5
0.0
−0.5
−1.0
−1.5
−2.0
−2.5
0
2
4
6
8
10
12
Educational screen time hours

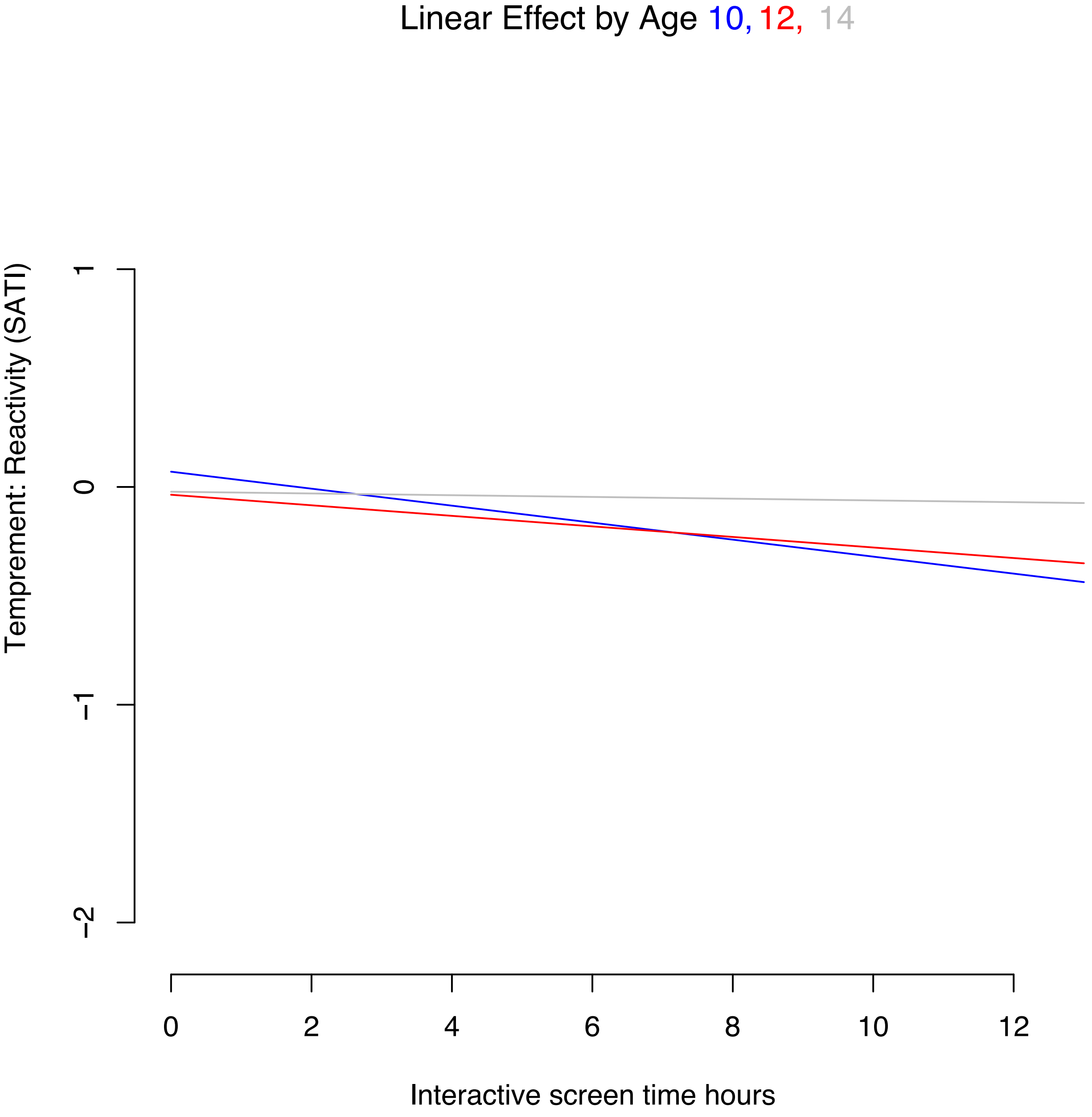

Linear Effect by Age 10, 12, 14
Temprement: Reactivity (SATI)
1
0
−1
−2
0
2
4
6
8
10
12
Interactive screen time hours

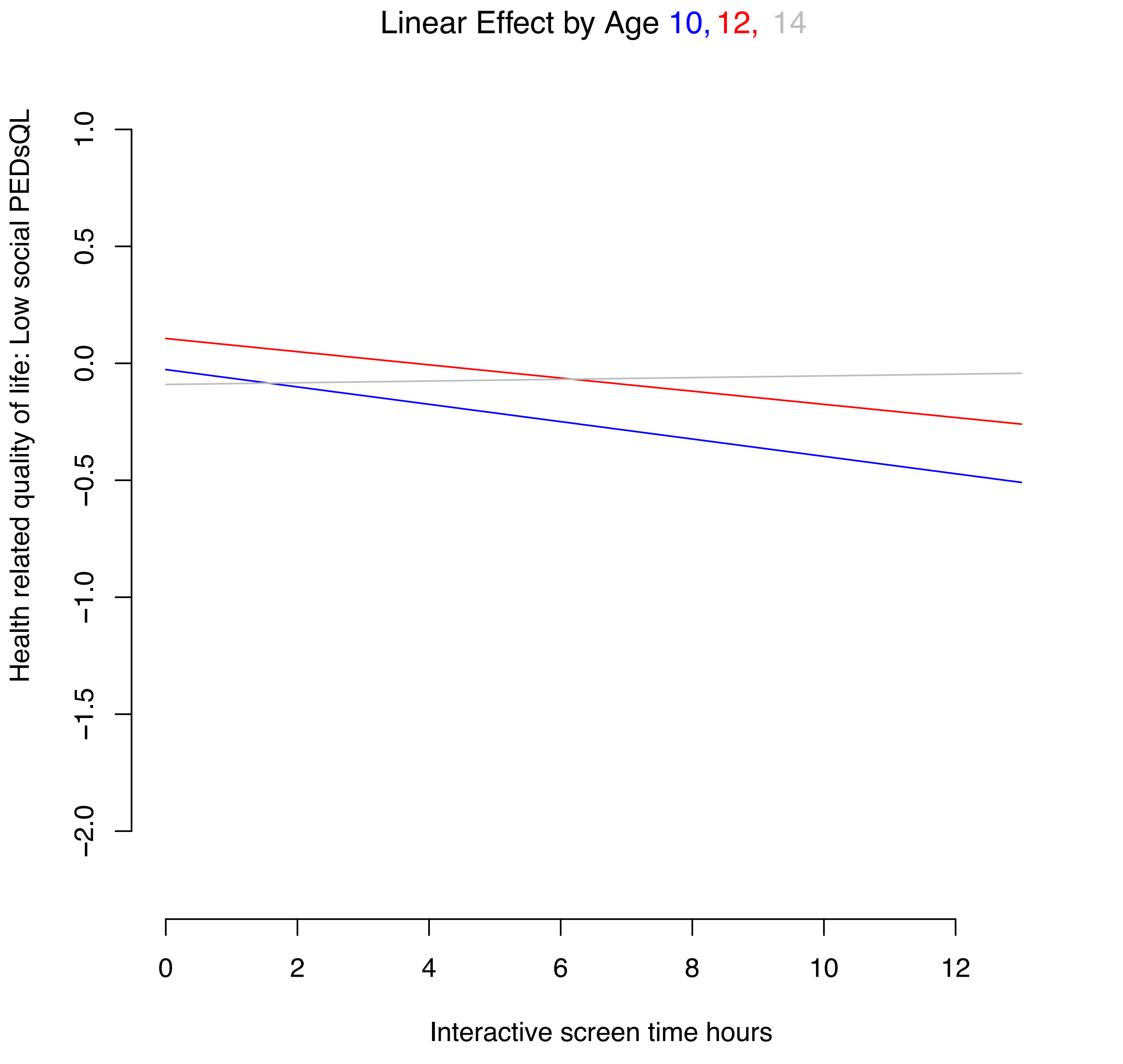

Linear Effect by Age 10, 12, 14
Health related quality of life: Low social PEDsQL
1.0
0.5
0.0
−0.5
−1.0
−1.5
−2.0
0
2
4
6
8
10
12
Interactive screen time hours

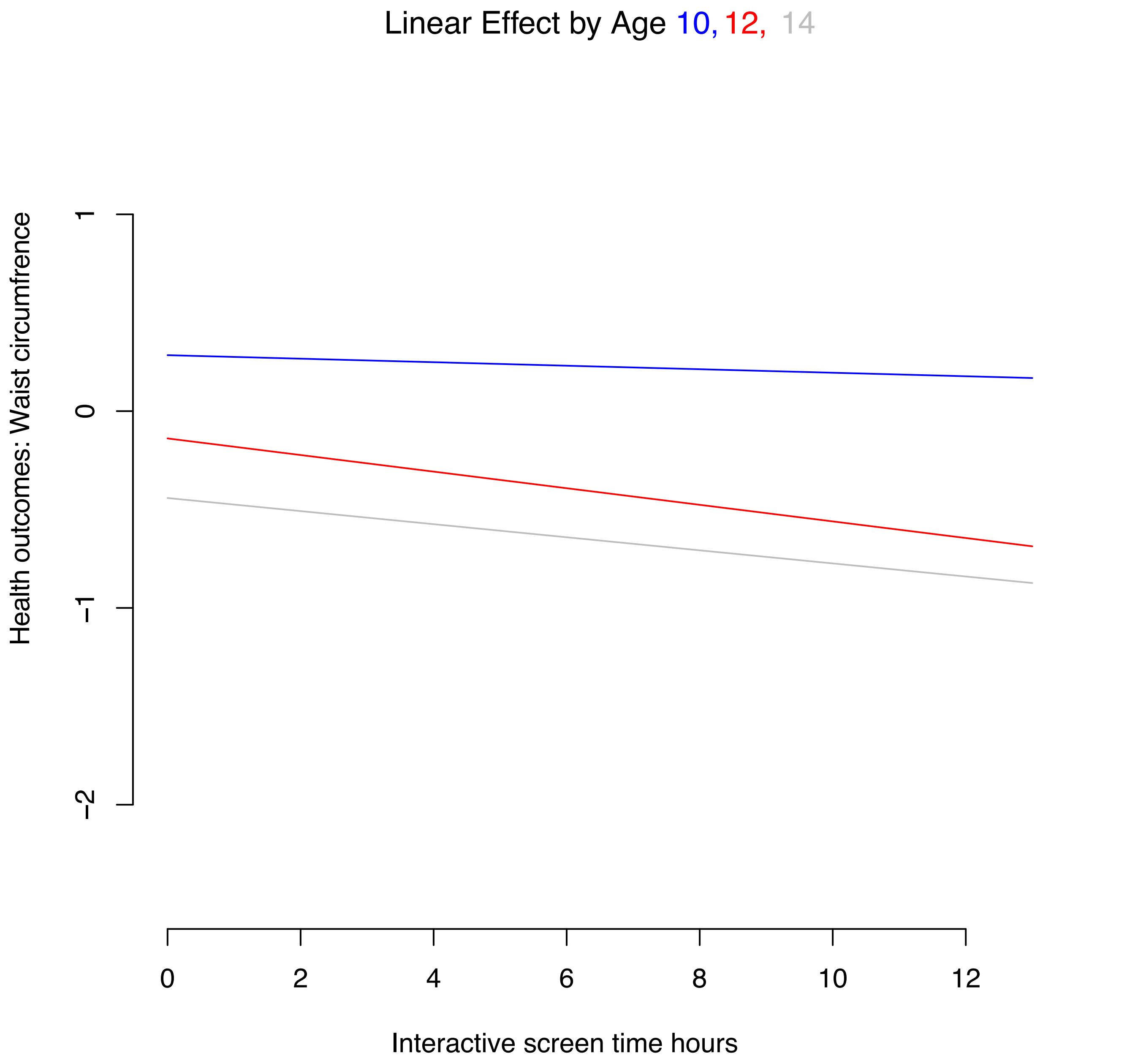

Linear Effect by Age 10, 12, 14
Health outcomes: Waist circumfrence
1
0
−1
−2
0
2
4
6
8
10
12
Interactive screen time hours

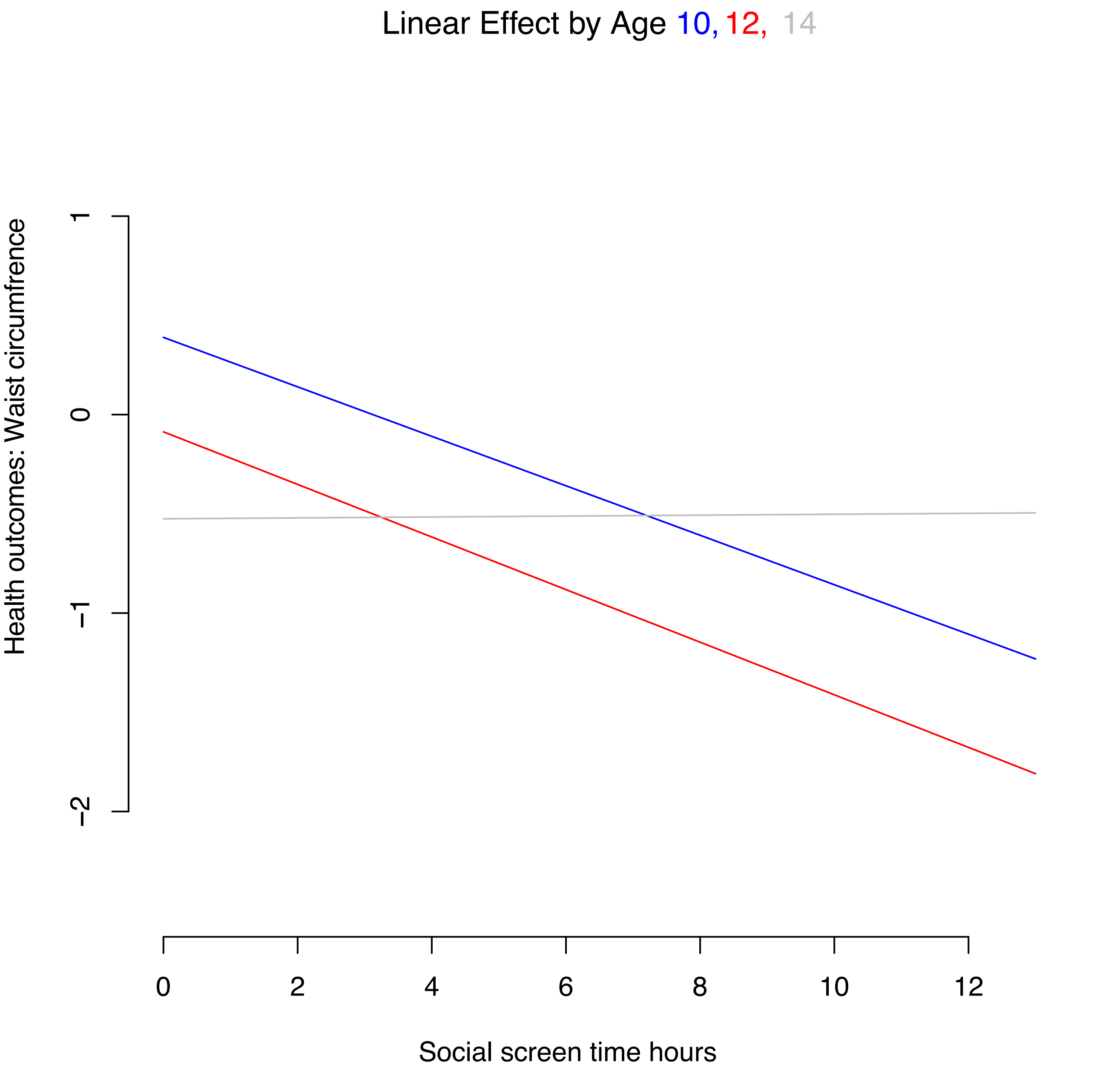

Linear Effect by Age 10, 12, 14
Health outcomes: Waist circumfrence
1
0
−1
−2
0
2
4
6
8
10
12
Social screen time hours

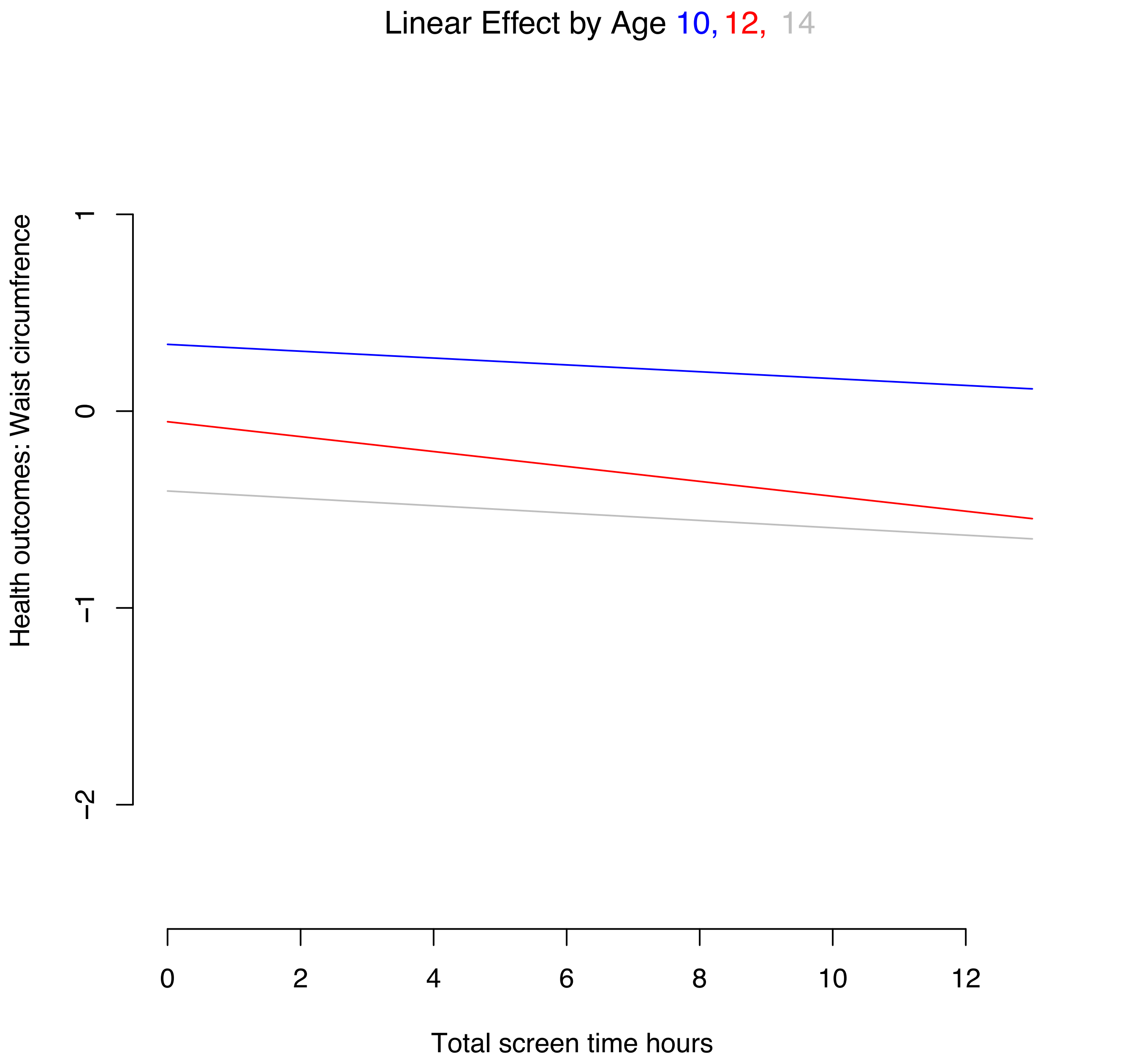

Linear Effect by Age 10, 12, 14
Health outcomes: Waist circumfrence
1
0
−1
−2
0
2
4
6
8
10
12
Total screen time hours

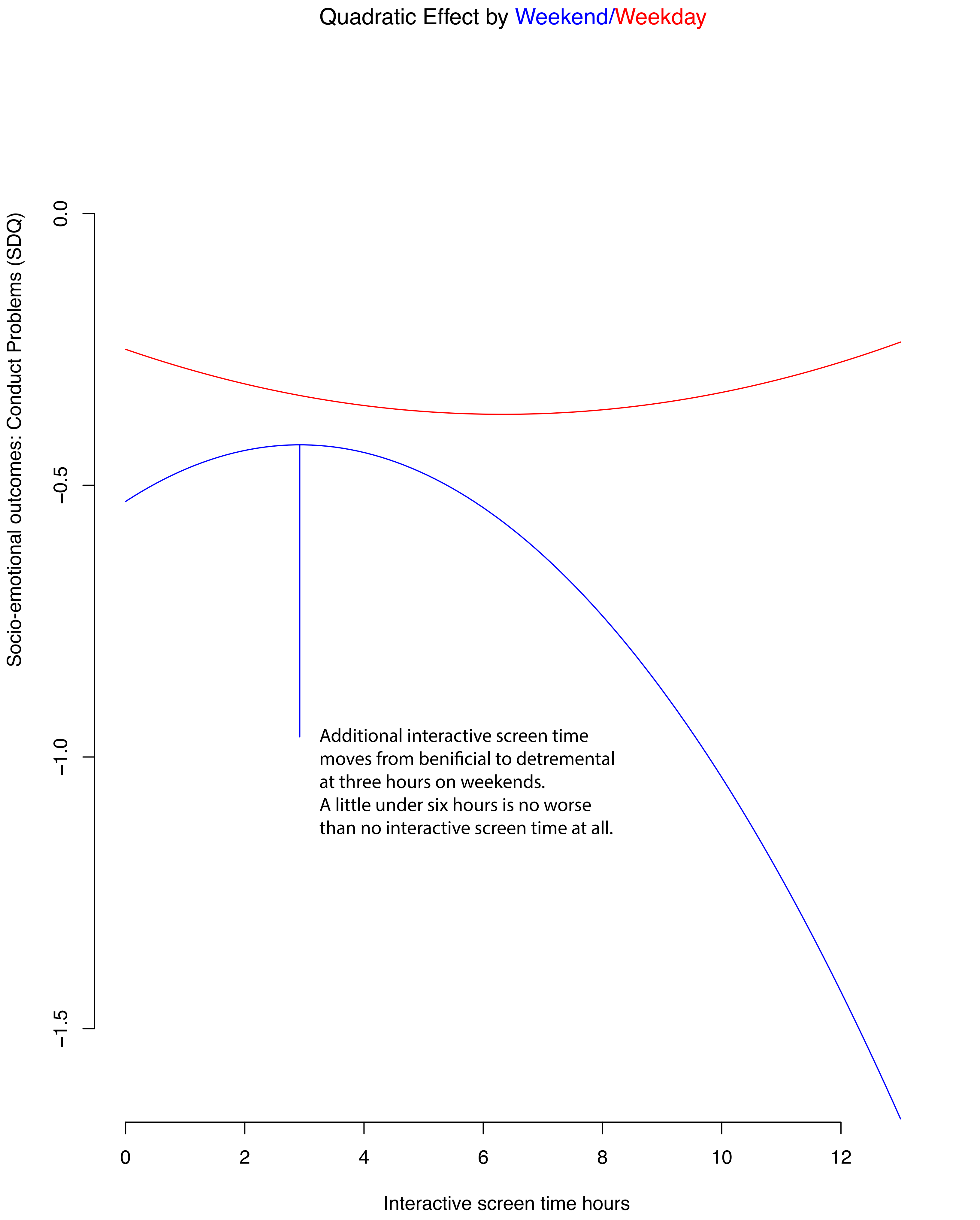
Quadratic Effect by Weekend/Weekday
Socio-emotional outcomes: Conduct Problems (SDQ)
0.0
−0.5
−1.0
−1.5
0
2
4
6
8
10
12
Interactive screen time hours
Additional interactive screen time
moves from benificial to detremental
at three hours on weekends.
A little under six hours is no worse
than no interactive screen time at all.

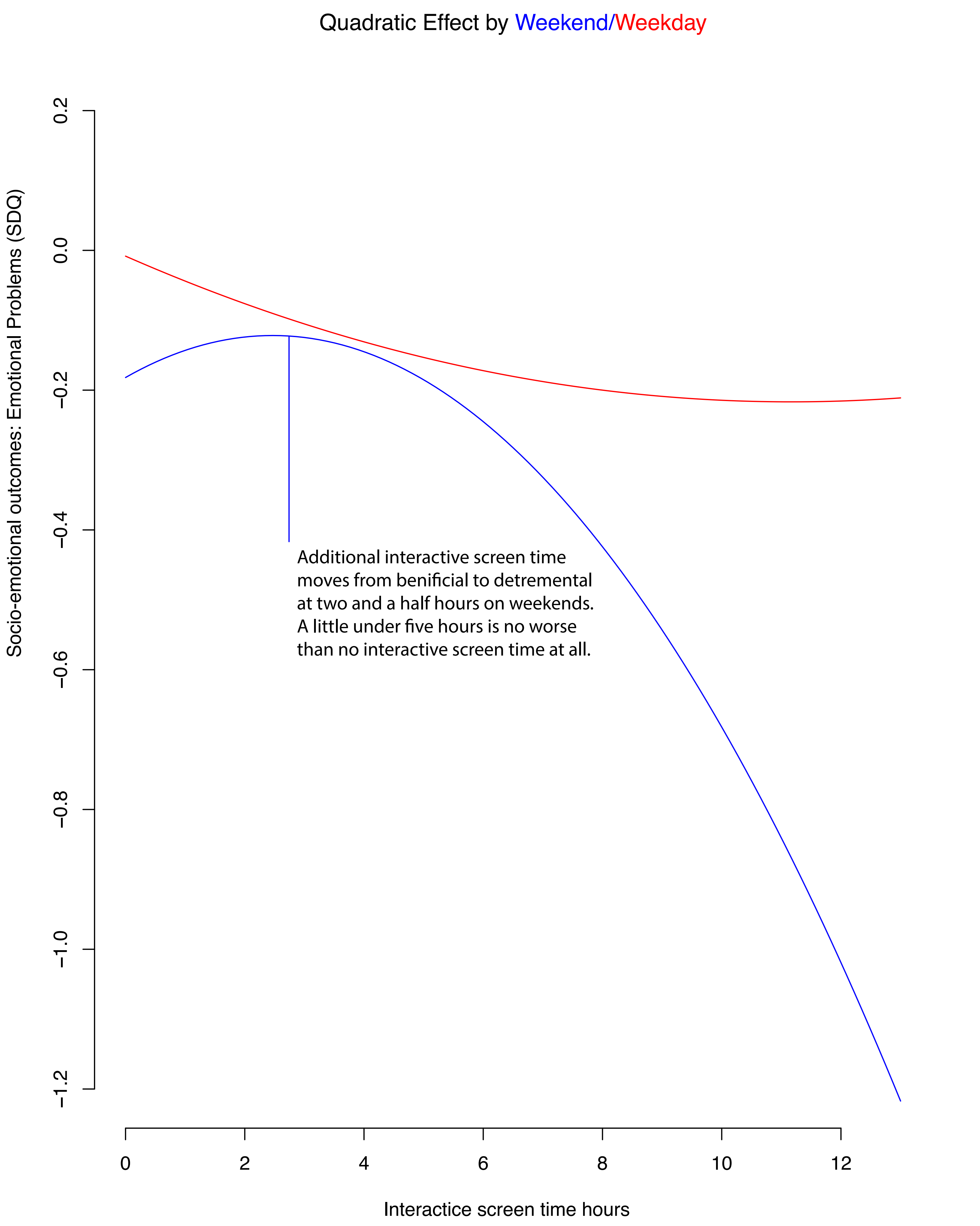
Quadratic Effect by Weekend/Weekday
Socio-emotional outcomes: Emotional Problems (SDQ)
0.2
0.0
−0.2
−0.4
−0.6
−0.8
−1.0
−1.2
Additional interactive screen time
moves from benificial to detremental
at two and a half hours on weekends.
A little under five hours is no worse
than no interactive screen time at all.
0
2
4
6
8
10
12
Interactice screen time hours

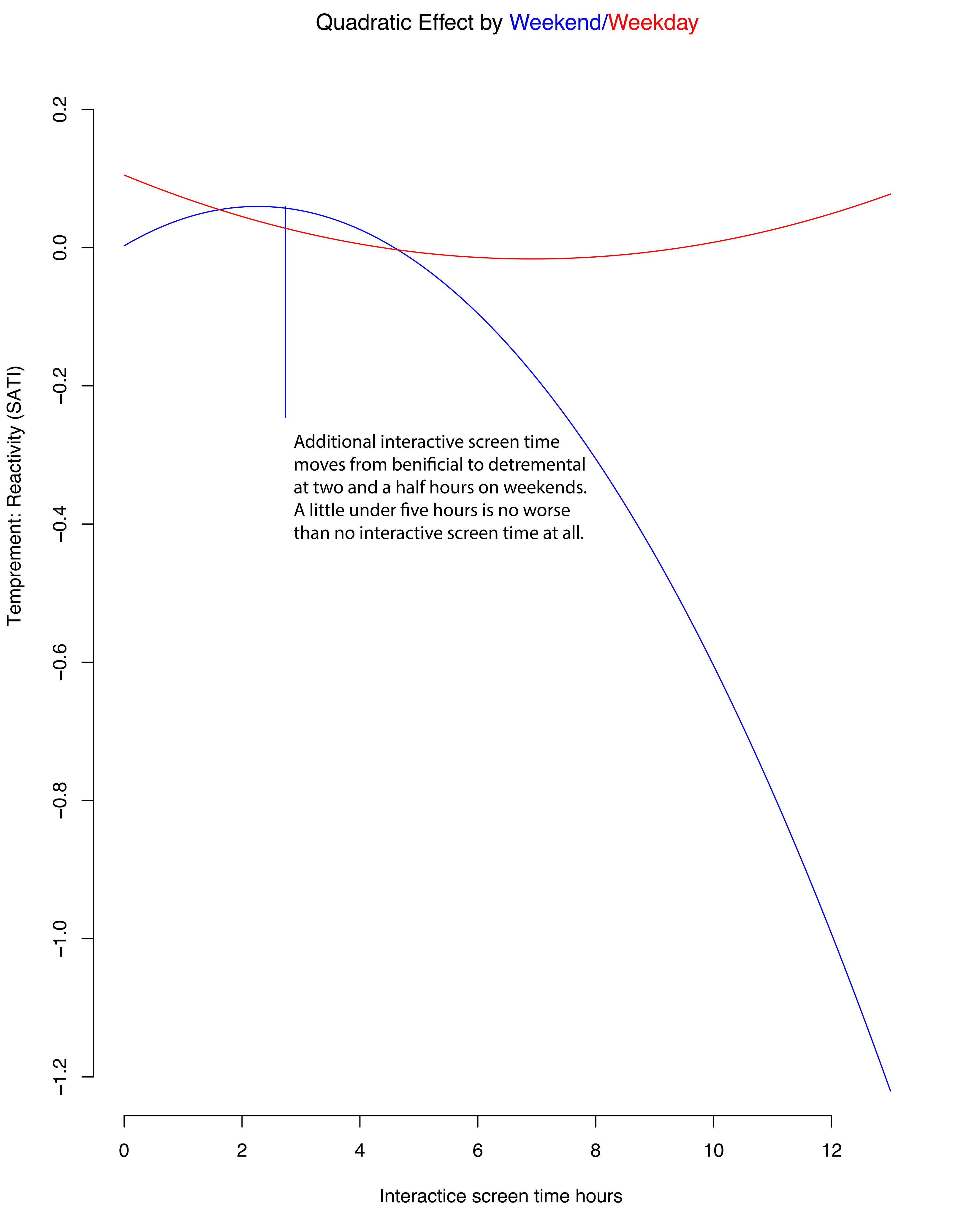
Quadratic Effect by Weekend/Weekday
Tempremement: Reactivity (SATI)
0.2
0.0
−0.2
−0.4
−0.6
−0.8
−1.0
−1.2
Additional interactive screen time
moves from benificial to detremental
at two and a half hours on weekends.
A little under five hours is no worse
than no interactive screen time at all.
0
2
4
6
8
10
12
Interactice screen time hours
