# Supplementary material for: Type of screen time moderates effects on outcomes in 4013 children: evidence from the Longitudinal Study of Australian Children
Source: Int J Behav Nutr Phys Act. 2019 Nov 29;16:117. doi: 10.1186/s12966-019-0881-7 (PMC6884886; doi:10.1186/s12966-019-0881-7)
Supplement: Supplementary file 7 — Additional file 7: Figure S4. Example plot of unadjusted linear and quadratic effects: social quality of life predicted by social screen time with original scale (left) and modified scale (right). [file 12966_2019_881_MOESM7_ESM.pdf]

Supplementary Figure 4: Example plot of unadjusted linear and quadratic effects: social quality of life predicted by social screen time with original scale (left) and modified scale (right)

Significant linear effects shown in yellow, and significant quadratic effect shown in black

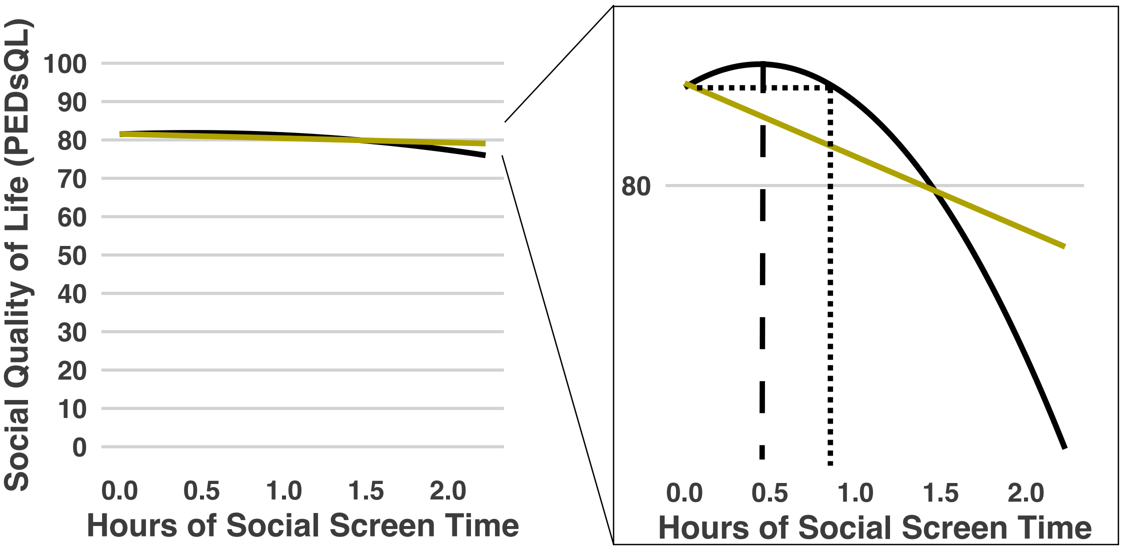

Dotted line indicates zero point: hours of screen time at which more screen time is worse than no screen time

Dashed line indicates turning point: hours of screen time at which more screen time becomes negative
